# Supplementary material for: Small Gastric Stromal Tumors: An Underestimated Risk
Source: Cancers (Basel). 2022 Dec 6;14(23):6008. doi: 10.3390/cancers14236008 (PMC9740305; doi:10.3390/cancers14236008)
Supplement: Supplementary file 1 [file cancers-14-06008-s001.zip › cancers-1972428-supplementary.pdf]

Supplementary Table S1: Clinicopathological characteristics and mutational information of 74 patients with small gastrointestinal stromal tumors. The risk classification was clarified according to the modified NIH criteria.

case No	Gender	Age	Tumor Size (cm)	Immunohistological	Risk classification	Mutations
P1	F	63	1.1	CD117(+);Dog1(+);CD34(+);SDHB(+);SMA(+);Desmin(-);S-100(partly+);Ki-67(2%+)	Low	PDGFRA c.A2525T p.D842V
P2	M	66	0.6	CD117(+);CD34(+);Desmin(-);Dog1(+);Ki-67(+1%);SMA(partly+);S-100(-)	Very Low	KIT c.1502_1503insTGCCTA p.S501delinsSAY
P3	M	53	0.8	SDHB(-);CD117(+);CD34(+);Desmin(-);Dog1(+);Ki-67(+);S-100(-)	Very Low	KIT c.C1652G p.P551R; c.T1679C p.V560A
P4	F	66	1.6	SDHB(+);CD117(+);CD34(+);Desmin(-);Dog1(+);Ki-67(5%+);SMA(-);S-100(-)	Low	KIT c.1733_1735del p.578_579del
P5	M	56	1.8	SDHB(-);CD117(+);CD34(+);Desmin(-);Dog1(+);Ki-67(+5%);SMA(partly+);S-100(-)	Low	KIT c.1675_1677del p.559_559del
P6	F	48	1.5	SDHB(+);CD117(+);CD34(+);Desmin(-);Dog1(+);Ki-67(+5%);SMA(-);S-100(-)	Very Low	KIT c.T1669C p.W557R
P7	M	30	1.5	SDHB(+);CD117(+);CD34(+);Desmin(-);Dog1(+);Ki-67(3%+);SMA(partly+);S-100(-)	Low	Not detected
P8	F	58	1.7	Dog-1(+);Ki-67(3%+);CD34(+);CD117(+);Desmin(-);S-100(-);SMA(-);SDHB(+)	Low	KIT c.1668_1679del p.556_560del
P9	F	49	1.1	SDHB(+);CD117(+);CD34(+);Desmin(-);Dog1(+);Ki-67(<1%+);SMA(partly+);S-100(-)	Very Low	BRAF c.T1799A p.V600E

P10	M	60	0.9	SDHB(-);CD117(+);CD34(+);Desmin(-);Dog1(+);Ki-67(-);SMA(-);S-100(-)	Very Low	BRAF c.T1799A p.V600E
P11	F	52	1.7	SDHB(+);CD117(+);CD34(partly+);Desmin(-);Ki-67(1%+);SMA(partly+);S-100(-)	Very Low	KIT c.T1679A p.V560D
P12	M	52	1.2	CD34(+);CD117(+);Dog1(+);SDHB(+);SMA(partly+);Desmin(-);S-100(-);Ki-67(<1%+)	Very Low	KIT c.1667_1681del p.556_561del
P13	M	46	1.5	SDHB(+);CD117(+);CD34(+);Desmin(-);Dog1(+);Ki-67(2%+);SMA(+);S-100(-)	Very Low	KIT c.1719_1739del p.573_580del
P14	M	48	1.8	Dog-1(+);Ki-67(5%+);CD34(+);CD117(+);Desmin(-);S-100(-);SMA(partly+);SDHB(+)	Low	KIT c.1502_1503insTGCCTA p.S501delinsSAY
P15	F	58	0.7	Dog-1(+);Ki-67(3%+);CD34(+);CD117(+);Desmin(-);S-100(-);SMA(-);SDHB(+)	Low	KIT c.T1679A p.V560D
P16	F	75	1	Dog-1(+);Ki-67(2%+);CD34(+);CD117(+);Desmin(-);S-100(-);SMA(-);SDHB(+)	Low	KIT c.T1727C p.L576P
P17	F	61	0.8	Dog-1(+);Ki-67(2%+);CD34(+);CD117(+);Desmin(-);S-100(-);SMA(-);SDHB(+)	Low	KIT c.1670_1675del p.557_559del
P18	F	47	0.6	Dog-1(+);Ki-67(1%+);CD34(+);CD117(+);Desmin(-);S-100(-);SMA(-);SDHB(+)	Very Low	KIT c.T1669C p.W557R
P19	M	61	1.2	Dog-1(+);Ki-67(5%+);CD34(+);CD117(+);Desmin(-);S-100(-);SMA(-);SDHB(+)	Very Low	KIT c.T1676A p.V559D

P20	M	50	0.5	Dog-1(+);Ki-67(3%+);CD34(+);CD117(+);Desmin(-);S-100(-);SMA(-)	Very Low	KIT c.1671_1673del p.557_558del
P21	F	70	0.7	SDHB(+);CD117(+);CD34(+);Desmin(-);Dog1(+)Ki-67(1%);SMA(-);S-100(-)	Very Low	KIT c.T2466A p.N822K
P22	F	53	1	CD34(+);CD117(+);Dog1(+);SMA(+);Desmin(partly +);SDHB(partly+);S-100(-);Ki-67(+)	Very Low	KIT c.1660_1674del p.554_558del
P23	F	66	1.5	SDHB(+);CD117(+);CD34(+);Desmin(-);Dog1(+);Ki-67(3%+);SMA(-);S-100(-);	Very Low	KIT c.1669_1670insTTC p.W557delinsFR
P24	F	69	1.5	SDHB(+);CD117(+);CD34(+);Desmin(-);Ki-67(10%+);SMA(-);S-100(-)	High	KIT c.T1676A p.V559D
P25	F	51	1.8	CD117(+);CD34(+);Desmin(-);Dog1(+);Ki-67(+10%);SMA(-);S-100(-)	Intermediate	KIT c.1502_1503insTGCCTA p.S501delinsSAY
P26	F	55	1.6	SDHB(+);CD117(+);CD34(+);Desmin(-);Dog1(+);Ki-67(4%+);S-100(-)	Intermediate	KIT c.1673_1693del p.558_565del
P27	F	49	0.8	SDHB(+);CD117(+);CD34(+);Desmin(-);Dog1(+);Ki-67(2%+);S-100(-)	Low	KIT c.1675_1677del p.559_559del
P28	F	48	1.7	CD117(+);CD34(+);Desmin(-);Dog1(+);Ki-67(3%+);SMA(-)	Low	KIT c.1667_1672del p.556_558del
P29	F	51	0.9	SDHB(+);CD117(+);CD34(partly+);Desmin(-);Dog1(+);Ki-67(1%+);S-100(-)	Very Low	KIT c.1733_1735del p.578_579del
P30	F	56	0.8	SDHB(+);CD117(+);CD34(+);Desmin(-);Dog1(+);Ki-67(+2%);S-100(-)	Very Low	KIT c.1667_1672del p.556_558del
P31	F	56	0.6	SDHB(+);CD117(+);CD34(+);Desmin(-);Dog1(+);Ki-67(<1%+);S-100(-)	Very Low	KIT c.1725_1726ins30 p.Q575_L576ins10

P32	F	50	0.6	SDHB(+);CD117(+);CD34(+);Desmin(-);Dog1(+);Ki-67(1%+);SMA(-);S-100(-)	Very Low	BRAF c.T1799A p.V600E
P33	M	61	2	CD117(+);CD34(+);Desmin(-);Dog1(+);SMA(-);S-100(-)	Very Low	KIT c.1716_1717insCCAACA p.D572delinsDPT
P34	M	54	0.7	CD117(+);CD34(+);Desmin(-);Dog1(+);SMA(-);S-100(-)	Very Low	BRAF c.T1799A p.V600E
P35	M	48	0.5	SDHB(+);CD117(+);CD34(+);Desmin(-);Ki-67(3%+);SMA(-);S-100(-)	Very Low	KIT c.T1676C p.V559A
P36	F	71	1.8	SDHB(+);CD117(+);CD34(+);Desmin(-);Dog1(+);Ki-67(5%+);S-100(-)	Low	KIT c.1716_1717insCCAACA p.D572delinsDPT
P37	F	59	1.9	CD117(+),CD34(+),DOG1(-),SMA(-),Desmin(-),S100(-),SDHB(+),Ki67(3%)	Very Low	KIT c.T1676A p.V559D
P38	M	51	1.1	CD117(+),CD34(+),DOG1(+),SMA(-),Desmin(-),S100(-),SDHB(-),Ki67(5%)	Very Low	KIT c.T1676A p.V559D
P39	F	73	1	CD117(+),CD34(+),DOG1(+),SMA(-),Desmin(-),S100(-),SDHB(-),Ki67(1%)	Very Low	KIT c.T1676A p.V559D
P40	M	54	0.8	CD117(+),CD34(+),DOG1(+),SMA(+),Desmin(-),S100(-),SDHB(+),Ki67(2%)	Very Low	KIT c.T1669A p.W557R
P41	F	64	1.5	CD117(+),CD34(+),DOG1(+),SMA(-),Desmin(-),S100(-),SDHB(-),Ki67(2%)	Very Low	KIT c.T1669A p.W557R
P42	F	58	0.6	CD117(+),CD34(+),DOG1(+),SMA(+),Desmin(+),S100(-),SDHB(+),Ki67(3%)	Very Low	BRAF c.T1799A p.V600E
P43	M	56	1.8	CD117(+),CD34(+),DOG1(+),SMA(-),Desmin(-),S100(-),SDHB(+),Ki67(10%)	Very Low	KIT c.C1352T p.S451F

P44	F	56	0.4	CD117(+),CD34(+),DOG1(+),SMA(-)Desmin(-),S100(-),SDHB(+),Ki67(1%)	Very Low	BRAF c.T1799A p.V600E
P45	F	49	1	CD117(+),CD34(+),DOG1(+),SMA(-),Desmin(-),S100(-),SDHB(+),Ki67(1%)	Very Low	PDGFRA c.A2543C p.N848T
P46	M	65	0.8	CD117(+),CD34(+),DOG1(+),SMA(-),Desmin(-),S100(-),SDHB(-),Ki67(1%)	Very Low	KIT c.T1669A p.W557R
P47	M	69	0.9	CD117(+),CD34(+),DOG1(+),SMA(+),Desmin(+),S100(-),SDHB(-),Ki67(1%)	Very Low	PDGFRA c.C2544A p.N848K
P48	M	60	0.8	CD117(+),CD34(+),DOG1(+),SMA(-),Desmin(-),S100(-),SDHB(-),Ki67(2%)	Very Low	KIT c.T1669A p.W557R
P49	M	41	1.2	CD117(-),CD34(+),DOG1(+),SMA(-),Desmin(-),S100(-),SDHB(-),Ki67(35%)	Intermediate	KIT c.T1669A p.W557R
P50	F	54	1	CD117(+),CD34(+),DOG1(+),SMA(-),Desmin(-),S100(-),SDHB(-),Ki67(5%)	Very Low	KIT c.T1676C p.V559A
P51	F	70	1.3	CD117(+),CD34(+),DOG1(+),SMA(-),Desmin(-),S100(-),SDHB(-),Ki67(1%)	Very Low	KIT c.T1676C p.V559A
P52	F	48	1	CD117(+),CD34(+),DOG1(+),SMA(-),Desmin(-),S100(-),SDHB(-),Ki67(1%)	Very Low	KIT c.C1352T p.S451F
P53	F	67	1.2	CD117(+),CD34(+),DOG1(+),SMA(-),Desmin(-),S100(-),SDHB(-),Ki67(1%)	Very Low	KIT c.T1676A p.V559D
P54	M	50	2	CD117(+),CD34(+),DOG1(+),SMA(-)Desmin(-),S100(-),SDHB(-),Ki67(1%)	Very Low	BRAF c.T1799A p.V600E
P55	F	69	1	CD117(+),CD34(+),DOG1(+),SMA(-),Desmin(-),S100(-),SDHB(-)	Very Low	KIT c.T1679A p.V560D
P56	M	70	1.5	CD117(+),CD34(+),DOG1(+),SMA(-)Desmin(-),S100(-),SDHB(-),Ki67(1%)	Very Low	BRAF c.T1799A p.V600E

P57	M	58	1.2	CD117(+),CD34(+),DOG1(+),SMA(-),Desmin(-),S100(-),SDHB(+),Ki67(2%)	Very Low	Not detected
P58	F	29	0.3	CD117(+),CD34(+),DOG1(+),SMA(+),Desmin(-),S100(-),SDHB(-),Ki67(6%)	Very Low	KIT c.T1669A p.W557R
P59	F	68	0.8	CD117(+),CD34(+),DOG1(+),SMA(-),Desmin(-),S100(-),SDHB(-),Ki67(2%)	Very Low	KIT c.A1672G p.K558E
P60	F	47	0.3	CD117(+),CD34(+),DOG1(+),SMA(+),Desmin(-),S100(-),SDHB(+)	Very Low	BRAF c.T1799A p.V600E
P61	F	62	0.6	CD117(+),CD34(+),DOG1(+),SMA(-),Desmin(-),S100(-),SDHB(-),Ki67(1%)	Very Low	KIT c.T1676A p.V559D
P62	F	40	0.5	CD117(+),CD34(+),DOG1(+),SMA(+),Desmin(+),S100(-),SDHB(+),Ki67(1%)	Very Low	PDGFRA c.1698_1712del p.S566_E571delinsR
P63	M	41	1	CD117(+),CD34(+),DOG1(+),SMA(+),Desmin(+),S100(-),SDHB(+),Ki67(1%)	Very Low	KIT c.T1669A p.W557R
P64	F	43	1.2	CD117(+),CD34(+),DOG1(+),SMA(-),Desmin(-),S100(-),SDHB(-),Ki67(<1%)	Very Low	KIT c.C1721T p.T574I
P65	M	50	1	CD117(+),CD34(+),DOG1(+),SMA(+),Desmin(+),S100(-),SDHB(-),Ki67(<1%)	Very Low	BRAF c.T1799A p.V600E
P66	F	31	0.5	CD117(+),CD34(+),DOG1(+),SMA(+),Desmin(-),S100(-),SDHB(+),Ki67(3%)	Very Low	BRAF c.T1799A p.V600E
P67	F	55	1.3	CD117(+),CD34(+),DOG1(+),SMA(-),Desmin(-),S100(-),SDHB(-),Ki67(3%)	Very Low	KIT c.C2063T p.S688L
P68	F	65	1.5	CD117(+),CD34(+),DOG1(+),SMA(-),Desmin(-),S100(-),SDHB(+),Ki67(2%)	Very Low	KIT c.T1676A p.V559D

P69	F	69	0.8	CD117(+),CD34(+),DOG1(+),SMA(-),Desmin(-),S100(-),SDHB(+),Ki67(3%)	Very Low	KIT c.C1652T p.P551L
P70	M	68	1.2	CD117(+),CD34(+),DOG1(+),SMA(-),Desmin(-),S100(-),SDHB(+),Ki67(8%)	Very Low	KIT c.A1924G p.K642E
P71	F	68	1.5	CD117(+),CD34(+),DOG1(+),SMA(-),Desmin(-),S100(-),SDHB(+),Ki67(3%)	Very Low	KIT c.T1679A p.V560D
P72	F	65	0.5	CD117(+),CD34(+),DOG1(+),SMA(-),Desmin(-),S100(-),SDHB(+),Ki67(1%)	Very Low	KIT c.T1676A p.V559D
P73	M	77	1.5	CD117(+),CD34(+),DOG1(+),SMA(-),Desmin(-),S100(-),SDHB(-),Ki67(1%)	Very Low	KIT c.G1675A p.V559I
P74	F	63	1	CD117(+),CD34(+),DOG1(+),SMA(-),Desmin(-),S100(-),SDHB(+),Ki67(2%)	Very Low	Not detected
